# LINGO-1 antibody ameliorates myelin impairment and spatial memory deficits in experimental autoimmune encephalomyelitis mice

**DOI:** 10.1038/srep14235

**Published:** 2015-09-18

**Authors:** Jun-Jun Sun, Qing-Guo Ren, Lin Xu, Zhi-Jun Zhang

**Affiliations:** 1 Department of Neuropsychiatry, Affiliated ZhongDa Hospital, School of Medicine, Southeast University, Nanjing 210009, Jiangsu, China; 2 Key Laboratory of Animal Models and Human Disease Mechanisms, Chinese Academy of Sciences & Yunnan Province, Kunming Institute of Zoology, Kunming 650223, Yunnan, China; 3 Graduate School of Chinese Academy of Sciences, Beijing 100049, China

## Abstract

More than 50% of multiple sclerosis patients develop cognitive impairment. However, the underlying mechanisms are still unclear, and there is no effective treatment. LINGO-1 (LRR and Ig domain containing NOGO receptor interacting protein 1) has been identified as an inhibitor of oligodendrocyte differentiation and myelination. Using the experimental autoimmune encephalomyelitis (EAE) mouse model, we assessed cognitive function at early and late stages of EAE, determined brain expression of myelin basic protein (MBP) and investigated whether the LINGO-1 antibody could restore deficits in learning and memory and ameliorate any loss of MBP. We found that deficits in learning and memory occurred in late EAE and identified decreased expression of MBP in the parahippocampal cortex (PHC) and fimbria-fornix. Moreover, the LINGO-1 antibody significantly improved learning and memory in EAE and partially restored MBP in PHC. Furthermore, the LINGO-1 antibody activated the AKT/mTOR signaling pathway regulating myelin growth. Our results suggest that demyelination in the PHC and fimbria-fornix might contribute to cognitive deficits and the LINGO-1 antibody could ameliorate these deficits by promoting myelin growth in the PHC. Our research demonstrates that LINGO-1 antagonism may be an effective approach to the treatment of the cognitive impairment of multiple sclerosis patients.

Multiple sclerosis (MS) is one of the most common demyelinating diseases of the central nervous system (CNS), and more than 50% of MS patients develop cognitive impairment, including abnormalities in information processing speed, attention, and memory^1^. These deficits detrimentally affect many aspects of daily life in MS patient populations, including the high frequency of unemployment^2^. Experimental autoimmune encephalomyelitis (EAE) is the most widely used model of MS. Consistent with the findings from MS investigations, the EAE model also produces spatial learning and memory deficits^3^,^4^,^5^.

Myelin has a specialized multilamellar structure and wraps around neuronal axons via the plasma membrane of oligodendrocytes in the CNS. It is an important structural and functional part of the CNS. It increases the velocity of transmission of action potentials, provides trophic support to the neuronal axons^6^,^7^, and maintains the long-term integrity of myelinated axons^8^. However, myelin is a fragile structure and is especially sensitive to many adverse factors including ischemia, hypoxia, toxins or inflammation^9^,^10^. Thus, the impairment of myelin is a prominent feature of many neurological diseases and complex neuropsychiatric disorders including MS and Alzheimer’s disease^11^,^12^,^13^. And, demyelination may be one of the factors that cause brain dysfunction, including cognitive impairment.

Many studies have demonstrated that there is a close relationship between myelin impairment and cognitive decline. MRI studies have indicated that myelin damage is associated with cognitive impairment in multiple sclerosis^14^,^15^,^16^. However, the non-invasive imaging investigations of MS mainly focus on the demyelination of white matter, but largely ignore demyelination in the gray matter. Alternatively, postmortem studies have demonstrated demyelination in the hippocampus of MS patients^17^,^18^, which is an important brain area associated with memory. However, cognitive testing was not possible in these postmortem studies. Consistent with postmortem clinical research, preclinical studies have also demonstrated demyelination in the hippocampus (CA1) in the EAE model^5^. However, to date, the neuropathological mechanisms involved in the cognitive impairment of the EAE model remain elusive.

Despite the high incidence of cognitive impairment in MS patients, the data indicate that most of the pharmacological symptomatic treatments for MS have no cognitive benefits, and there is no effective treatment aimed at recovering the cognitive impairment^19^. LINGO-1 (Leucine rich repeat and Ig domain containing NOGO receptor interacting protein 1) is an important transmembrane protein that is specifically expressed in oligodendrocytes and neurons in the CNS; it is a key inhibitor of oligodendrocyte precursor cells (OPCs) differentiation and myelination^20^. Attenuation of LINGO-1 function with the LINGO-1 antibody facilitates OPCs differentiation and myelination *in vitro*, whereas overexpression of LINGO-1 inhibited the differentiation of OPCs and myelination^21^. Consistent with the observations *in vitro*, LINGO-1 knock-out mice show early-onset CNS myelination compared with wild-type^21^ and transgenic mice overexpressing the full length LINGO-1 display a reduction in CNS myelination^22^. Further research by Mi *et al.* (2007) demonstrates that the LINGO-1 antagonist promotes spinal cord remyelination and functional recovery in EAE mice^23^. These studies provide the evidence to confirm that antagonism of LINGO-1 is one of promising approaches for the treatment of demyelinating diseases. It has been well demonstrated that the LINGO-1 antibody promotes remyelination; however, whether the LINGO-1 antibody could effectively restore the cognitive impairment in EAE mice is still unknown.

This research indicated that the EAE mice display impairment of spatial memory as well as demyelination in the parahippocampal cortex (PHC) and fimbria-fornix in the late stages of the disease. After the systemic administration of the LINGO-1 antibody, the memory impairment was restored and remyelination in the PHC was observed. Here, our research indicated that demyelination in the PHC may cause the spatial learning and memory impairment in EAE mice. Importantly, our results demonstrated that the therapeutic LINGO-1 antibody produced significant beneficial effects in the murine model of MS. Thus, the LINGO-1 antibody may be an effective drug for ameliorating the cognitive impairment of demyelinating diseases in the CNS.

## Results

### Impairment of learning and memory in EAE mice

**Figure 1:**
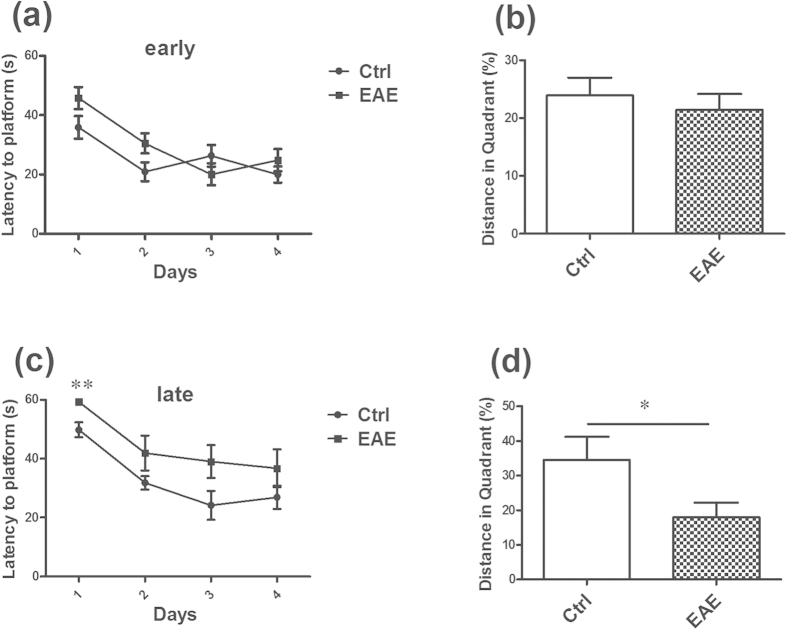
Impairment of special learning and memory in EAE mice in the late stage of disease. (**a**,**b**) It displayed no obvious cognitive decline in the EAE mice in the early stage. (**c**) In the late stage, the latency to finding the hidden platform in the EAE mice was longer than in the control mice, especially in the first day. (**d**) On the detecting day, the percentage of the distance in the target quadrant is significantly shorter than that of controls. *Denotes statistical significance compared with controls (P < 0.05). **Denotes statistical significance compared with controls (P < 0.01).

In our research, we performed the elevated plus maze (EPM), open field test, sucrose preference test and Morris water maze test (MWT) in both the early (day 13) and late (day 55) stages of EAE. After one batch of the behavior tests, the EAE mice and age-matched controls were killed. The results showed that the EAE mice have no anxiety and depression-like behavior in the two periods (Supplementary Figs S1–S3 online). It also displayed no significantly cognitive decline in the EAE mice in the early stage (Fig. 1a,b). However, in the late stage, the performance of the EAE mice in MWT was significantly worse compared with the control animals. The latency to the hidden platform in the EAE mice was longer than in the control mice, especially in the first day (Fig. 1c). Furthermore, on the detecting day, a defect in the spatial memory test is observed in the EAE mice, and the percentage of the distance in the target quadrant is significantly shorter than that of controls (Fig. 1d).

### The main impairment of the brain regions in EAE mice

**Figure 2:**
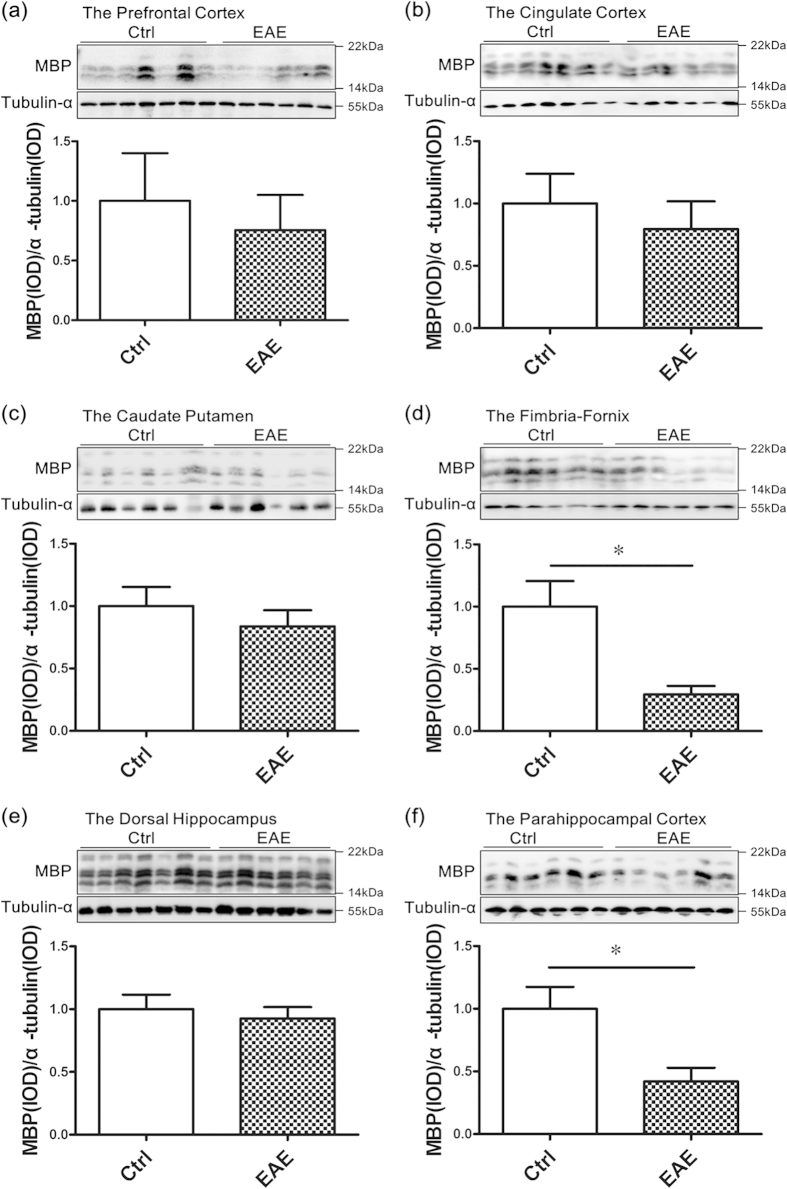
Demyeliantion in the parahippocampal cortex and fimbria-fornix. Western blot analysis showed that there was a significant decrease in the MBP level in EAE mice compared with controls, mainly in the PHC (**f**) and fimbria-fornix (**d**). No significant changes were found between the EAE and control groups in the prefrontal cortex (**a**), cingulate (**b**), caudate putamen (**c**) and dorsal hippocampus (**e**). *Denotes statistical significance compared with controls (P < 0.05).

In the research, we determined the myelin-associated protein (myelin basic protein) in six-different brain regions that are associated with spatial learning and memory: the prefrontal cortex, cingulate, caudate putamen (striatum), fimbria-fornix, dorsal hippocampus and PHC. The results showed that there was a significant decrease of myelin basic protein (MBP) in the PHC (Fig. 2f) and fimbria-fornix (Fig. 2d) of EAE mice. No significant changes were found between the EAE and control groups in other brain regions, including the prefrontal cortex, cingulate, caudate putamen (striatum), and dorsal hippocampus (Fig. 2a–c,e).

### The LINGO-1 antibody restores the impairment of spatial learning and memory in EAE mice

**Figure 3:**
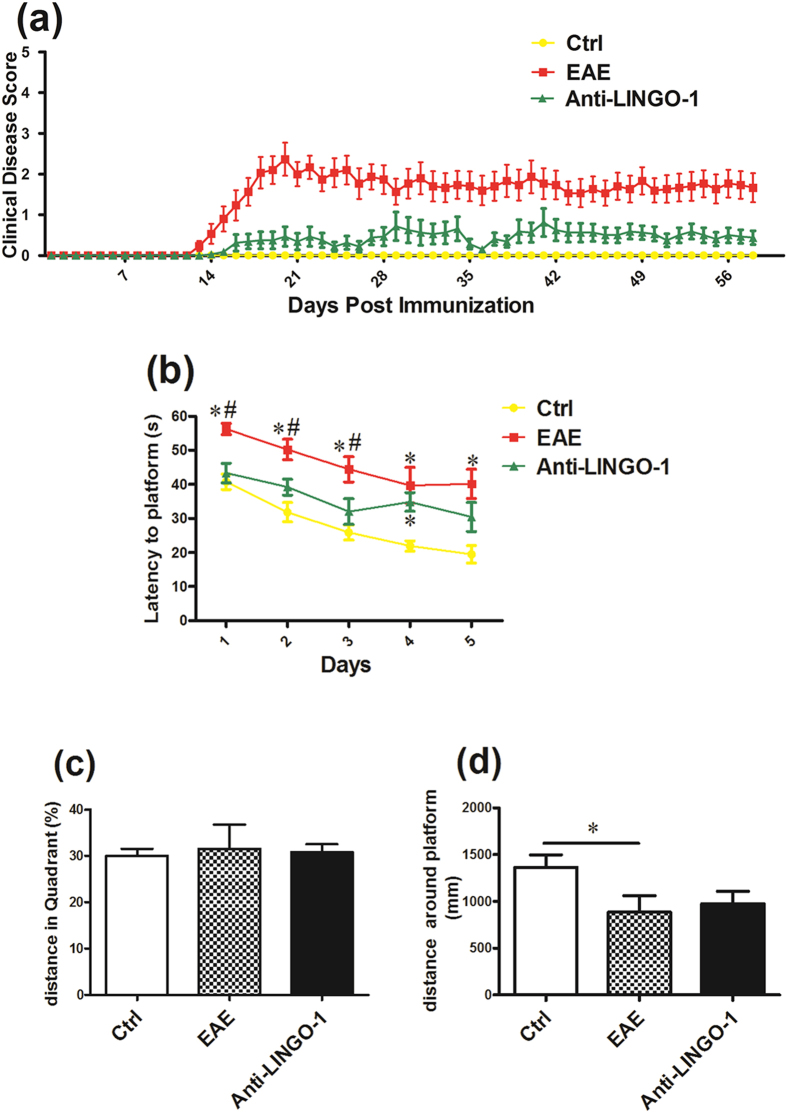
LINGO-1 antibody restores the impairment of spatial learning and memory in EAE mice(**a**) LINGO-1 antibody significantly reduced the EAE clinical scores (P < 0.05). (**b**) In the MWM, the mean escape latency of the EAE mice was significantly longer than the controls, and Anti-LINGO-1reduced the latency to the platform in treated EAE mice. (**c**) On the sixth day, there was no difference in the percent distance of the target quadrant among the three groups. (**d**) Further we evaluated the distance around the platform 10 cm and found that the distance covered by the EAE mice was significantly less than that of the control mice, whereas the LINGO-1 antibody-treated EAE mice covered more distance around the target quadrant than the EAE mice, but this difference was not significant. *Denotes statistical significance compared with controls (P < 0.05). ^#^Denotes statistical significance compared with EAE mice without treatment (P < 0.05).

In the research, the LINGO-1 antibody was administered in the EAE treated group from day 14 after MOG immunization, when the mice began to exhibit clinical symptoms. During the period of six-week treatment, we found that the LINGO-1 antibody significantly reduced the clinical scores (Fig. 3a). The Morris water maze was tested from day 53 to day 58, and the mice underwent five days of training and one day of detecting. As shown in the results, the mean escape latency of the EAE mice was significantly longer than the controls, whereas that of the EAE mice with systemic administration of the LINGO-1 antibody was significantly shorter compared with the mice that were not treated (Fig. 3b). On the sixth day, the platform was removed, and the probe trial was conducted. There was no difference in the percent distance of the target quadrant among the three groups (Fig. 3c). However, we further evaluated the distance around the platform. The results showed that the distance covered by the EAE mice was significantly less than that of the control mice, whereas the LINGO-1 antibody-treated EAE mice covered more distance around the platform than the EAE mice, but this difference was not significant (Fig. 3d).

### LINGO-1 antibody promotes the PHC remyelination in EAE mice

**Figure 4:**
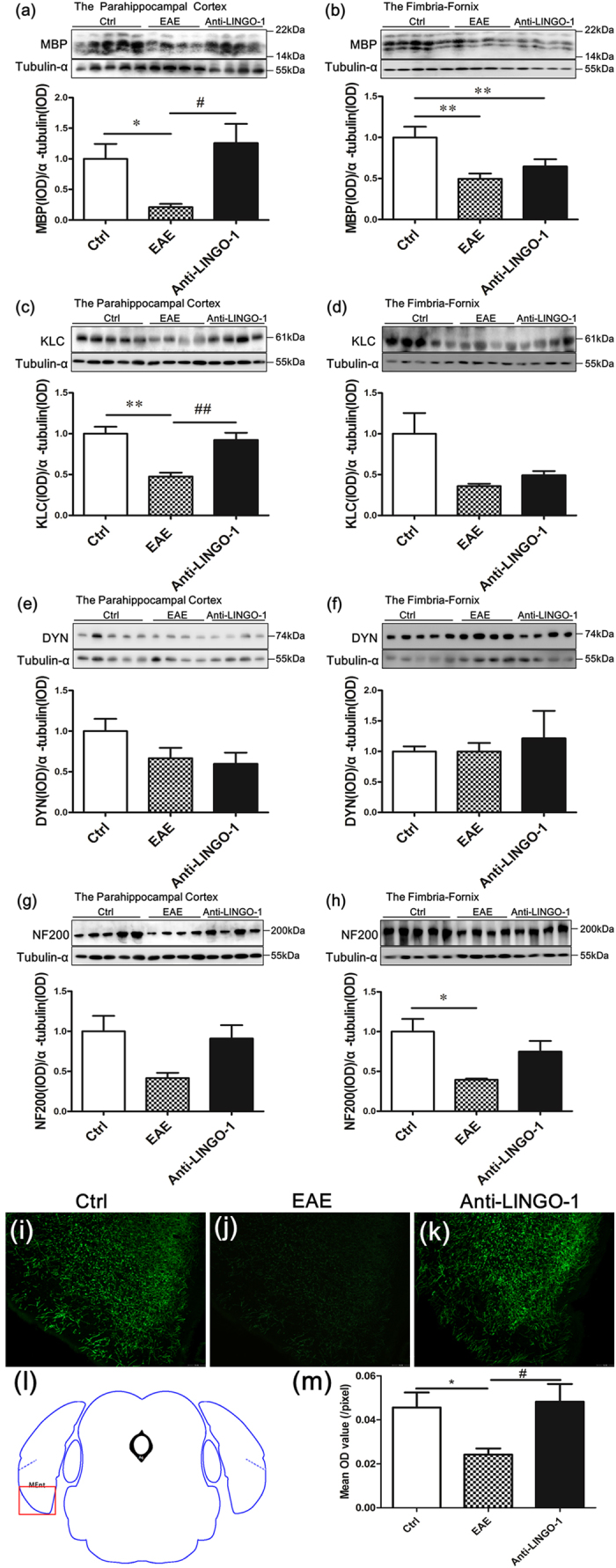
LINGO-1 antibody promotes parahippocampal cortex (PHC) remyelination in EAE mice. (**a**,**b**) The EAE mice exhibited demyelination in the PHC and fimbria-fornix. After a six-week treatment with the LINGO-1 antibody, the level of MBP was significantly increased in the PHC, but no significant change was observed in the fimbria-fornix. (**c**) We found a severe reduction in the expression of kinesin light chain (KLC), in the PHC of the EAE mice compared with controls, and the KLC expression was restored in the anti-LINGO-1 treated mice. (**e**)No significant differences were found for dynein (DYN), in the PHC of the EAE mice and the LINGO-1 antibody treated mice. (**g**) A reduction in the expression of neurofilament 200 (NF200), was detected in the EAE mice, and a slight increase was observed in the LINGO-1 antibody-treated mice. (**d**,**h**) KLC/NF200 expression was also decreased in the fimbria-fornix of the EAE but was not restored to normal levels in the EAE mice treated with the LINGO-1 antibody. (**f**) There was no significant difference in the expression of DYN among the three groups of mice. (**i**–**k**) Immunohistochemical staining for MBP in the three groups (**l**) Medial entorhinal cortex (MEnt) is an important subregion of the parahippocampal cortex. Schematic diagram display the scope we get the image in the microscope. (**j**) One-way ANOVA with least significance difference (LSD) test was used to determine statistical significance of MBP. The mean OD value was decline in the EAE mice and after LINGO-1 treatment, it was increased. n(Ctrl) = 3, n(EAE) = 4, n(Anti-LINGO-1) = 3. *Denotes statistical significance compared with controls (P < 0.05). **Denotes statistical significance compared with controls (P < 0.01). ^#^Denotes statistical significance compared with EAE mice without treatment (P < 0.05). ^##^Denotes statistical significance compared with EAE mice without treatment (P < 0.01). Scale bar, 100 μm.

As shown above, the EAE mice exhibited demyelination in the PHC and fimbria-fornix. After a six-week treatment with the LINGO-1 antibody, the level of MBP was significantly increased in the PHC (Fig. 4a), but no significant change was observed in the fimbria-fornix (Fig. 4b). At the same time, we detected proteins associated with the function of axons. We found a severe reduction in the expression of kinesin light chain (KLC), a functional protein of anterograde transport^24^, in the PHC of the EAE mice compared with that of controls, and the KLC expression was restored in the LINGO-1 antibody-treated mice (Fig. 4c). However, no significant differences were found for dynein (DYN), a protein associated with reversed transport^24^, in the PHC of the EAE mice and the LINGO-1 antibody treated mice (Fig. 4e). A reduction of neurofilament 200 (NF200), the heavy neurofilament of axons, was detected in the EAE mice, and a slight increase was observed in the LINGO-1 antibody-treated mice, but no significant difference was found (Fig. 4g). Furthermore, we also detected the levels of these proteins in the fimbria-fornix in the EAE mice. As shown in the results, KLC/NF200 expression was also decreased in the fimbria-fornix of the EAE but was not restored in the EAE mice treated with the LINGO-1 antibody (Fig. 4d,h). Similarly, there was no significant difference in the expression of DYN among the three groups of mice in the fimbria-fornix (Fig. 4f). Consistent with the results of western blot, in immunofluorescence staining, we also found decreased expression of MBP in the medial entorhinal cortex (an important subregion of the PHC) in the EAE mice and LINGO-1 antibody could restore decreased level of MBP (Fig. 4i–k).

### The possible mechanisms for the effect of the LINGO-1 antibody on remyelination

**Figure 5:**
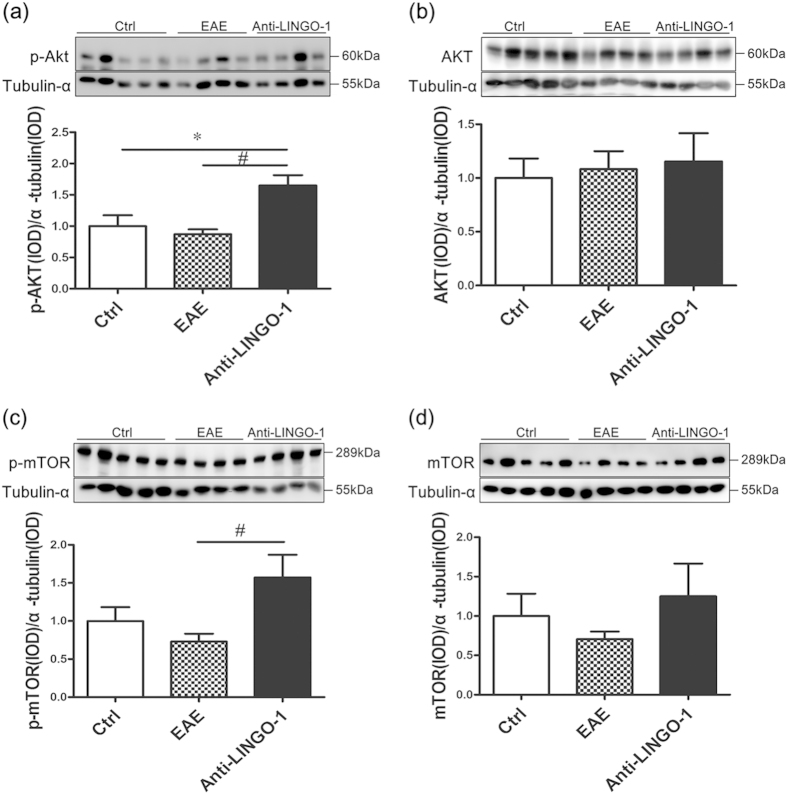
LINGO-1 antibody promotes remyelination by activating the PI3K/AKT/m-TOR signaling pathway(a) Compared to the control group, we also did not observe any obvious differences in the levels of Ser473-phosphorylated AKT in the EAE mice. However, compared to the EAE group, treatment with the LINGO-1 antibody clearly increased the levels of the Ser473-phosphorylated AKT. (**c**) Similar results were observed for p-mTOR. The level of p-mTOR was increased in the LINGO-1 antibody treated group. (**b,d**) No obvious differences were observed in the total levels of AKT and m-TOR among the three groups. *Denotes statistical significance compared with controls (P < 0.05). ^#^Denotes statistical significance compared with EAE mice without treatment (P < 0.05).

Many studies have demonstrated that the PI3K/AKT/m-TOR signaling pathway is involved in regulating myelination^25^,^26^,^27^,^28^,^29^. To detect whether the LINGO-1 antibody promotes remyelination by activating the PI3K/AKT/m-TOR signaling pathway, we measured the total level and the activity-dependent Ser473-phosphorylated level of AKT and the Ser2448-phosphorylated level of m-TOR in the PHC extracts. No obvious differences were observed in the total levels of AKT and m-TOR among the three groups (Fig. 5b,d). Compared to the control group, we also did not observe any obvious differences in the levels of Ser473-phosphorylated AKT (representing the activated form of the kinase) in the EAE mice (Fig. 5a). However, compared to the EAE group, LINGO-1 antibody clearly increased the levels of the Ser473-phosphorylated AKT (Fig. 5a). Similar results were observed for p-mTOR, and the ser2448-phoshorylated m-TOR was increased after LINGO-1 antibody treatment (Fig. 5c).

## Discussion

In the present study, we found that deficits in learning and memory occurred in the late stages of the disease rather than in the early stages. During the course of the disease, the EAE mice did not display anxiety and depression-like behaviors. We determined MBP in different brain regions and found results consistent with demyelination in the PHC and fimbria-fornix, which are involved in memory acquisition and maintenance. In the same regions, we also found significantly decreased levels of key structural and functional proteins in neuronal axons in the CNS. Moreover, the LINGO-1 antibody, which is thought to promote myelin restoration, significantly improved learning and memory in the EAE mice along with an increased expression of MBP and neural structural/functional proteins in PHC. Furthermore, the LINGO-1 antibody activated the AKT/mTOR signaling pathway, which is a key pathway regulating myelin growth. Our results suggest that demyelination in the PHC and fimbria-fornix might contribute to learning and memory deficits and the LINGO-1 antibody could ameliorate these deficits by promoting myelin growth in the PHC. The AKT/mTOR signaling pathway might be involved in this process. One important implication of these observations is that the LINGO-1 antibody could exert a neuroprotective effect that is effective for treating the cognitive impairment of MS.

Cognition is a complex brain function. Many studies have demonstrated that various brain regions, including the prefrontal cortex, cingulate, caudate putamen, fornix, hippocampus and PHC, are involved in memory formation; impairment in these brain regions may lead to memory deficits. In multiple sclerosis, the PHC exhibits lesions. A clinical study used 11C-PK11195 PET to evaluate microglial activation in the cortical gray matter in MS and showed microglial activation in the PHC which the authors suggest to be an indirect indicator of demyelination^30^. Furthermore, another group, using high-field 3.0 T MRI, three-dimensional T1-FSPGR, showed that the PHC displayed prominent cortical thinning in relapsing-remitting multiple sclerosis (RRMS) patients compared with healthy subjects and indicated substantial damage to the PHC in MS^31^. The fornix is the major hippocampal output of white matter fiber tracts, and is another key region that is associated with spatial learning and memory. A post-mortem study detected white lesions in the fornix in MS^32^. Consistent with those findings, MRI also demonstrates demyelination in the fornix in MS^15^. A further study used imaging to demonstrate that decreased fractional anisotropy (FA) in the fornix was correlated with both lower fMRI activity in the hippocampus and worse memory performance in MS; that study indicated that damage to the fornix can lead to the memory impairment in MS patients^33^. In EAE model, histopathology demonstrated demyelination in the fornix^34^. Thus both clinical and preclinical evidence indicate that lesions occur in the fornix in MS patients or EAE. Consistent with previous studies, we also found that there was a significant decrease in the levels of MBP in the PHC and fornix in EAE mice compared to controls. Combining the findings from our study and other studies, we suggest that demyelination in the PHC and fornix may contribute to impairment of spatial learning and memory in EAE mice.

We have used the MWM to determine these cognitive impairments in the EAE model. The MWM is robust and reliable test to determine spatial learning and memory, and correlates well with hippocampal function^35^. While it relies on the ability of animals to swim to search for a hidden platform, it is relatively insensitive to motor activity or swimming speed^35^. Thus, while the EAE mice do have a physical disability which is exhibited as a decrease in swimming speed in the MWM, this should not influence the cognitive performance. We have demonstrated that the spontaneous locomotor speed of EAE is similar to the control in the open field tests (Supplementary Information S1) and therefore we speculated the changes are interpreted as consequences of a cognitive deficit.

However, unexpectedly, a significant decrease in MBP was not detected in the hippocampus in our research, which was inconsistent with previous results^5^,^17^. It is apparent that the methods used to produce the EAE models are different from previous studies, and the average clinical score in the EAE mice in our study was lower than theirs. Based on previous study and our research, we infer that in EAE mice, the PHC and fornix might be more vulnerable to demyelination than the hippocampus. Additionally, while we detected the levels of MBP in the entire dorsal hippocampus, the previous study determined the content of MBP solely in the CA1 region. Thus there may be differential effects in different substructures of the hippocampus. Furthermore, we acknowledge that the western blot variance within each group is fairly high, and are aware that this may mean we underestimate (with false-negatives) the significance of differences between groups.

In the CNS, myelin not only exists in the white matter but is also present in the gray matter. Myelination is an important component of brain maturation, which is essential for normal brain function and enables higher order motor, sensory, and cognitive functioning^13^,^36^,^37^. Myelin provides critical functional support for axons in the CNS, and is involved in trophic support, maintenance of axon integrity, reduction of ATP consumption and rapid transmission of action potentials^6^,^7^,^8^,^38^. Recent studies have demonstrated that myelin damage (from myelin protein mutants, inflammation, or toxins) may cause axonal degeneration and functional disorders in the CNS^8^,^9^. In the present study, we not only observed demyelination but also discovered axonal degeneration and low expression of functional proteins (such as axoplasmic transport related proteins) in the same region (the PHC). Our results suggest that myelin damage could also lead to axonal impairment, which is consistent with previous studies^17^. The mechanism of axonal impairment caused by myelin damage needs further investigation. It is thought that the mechanisms may also involve insufficient axonal energy support.

In 2010, Nave suggested that myelin damage may lead to cognitive impairment^8^. Many studies have used imaging analysis to investigate the relationship between demyelination and cognitive impairment, and their results indicate that demyelination in the white matter may cause cognitive abnormalities. In our study, we determined MBP in different brain regions and observed deficits in the PHC and fornix in EAE mice, consistent with demyelination that may underlie a decline in spatial learning and memory ability. We also found that the LINGO-1 antibody, which promotes remyelination in the EAE mouse^23^, ameliorates the deficits of spatial learning and memory as well as myelin damage in the PHC. We also provide evidence that remyelination may be an effective approach to treating the decline in cognitive impairment in MS.

The LINGO-1 antibody is effective as a treatment of EAE due to its potency, selectivity, and duration of treatment effects based on its long serum half-life^39^. Although antibodies used as drugs to target antigens in the CNS has been challenging due to low CNS penetration after systemic administration, a study in 2011 demonstrated that the LINGO-1 antibody can cross the damaged blood brain barrier in EAE mice and that the small percentage of the LINGO-1 antibody that reaches the CNS is sufficient to elicit myelin repair^39^. Previous studies have reported that systemic LINGO-1 antibody administration promotes spinal cord remyelination and axonal integrity and decreases the clinical scores in EAE mice^23^,^39^. Consistent with previous studies, we also found that the LINGO-1 antibody decreases the clinical scores and ameliorates markers of demyelination and axon damage in the PHC in EAE mice. Our results demonstrate the potential for the LINGO-1 antibody in treatment of the cognitive impairment of MS.

It is conceivable that the Anti-LINGO-1 exerts its effect through an anti-inflammatory action. However, a previous report^23^ (Mi *et al.*) has demonstrated that LINGO-1 deficiency has no effect on proliferation of, cytokine release from, or encephalogenicity of T cells. Furthermore, they also report anti-LINGO-1 antibody treatment showed no difference from control in markers of macrophages, microglia or T-cells in EAE spinal cord. This treatment was also reported to have no effect on the behavioral onset of EAE. Together with our results, we considered that LINGO-1 antibody exerts its effect through remyelination.

Although substantial evidence has demonstrated that the LINGO-1 antibody promotes remyelination, the underlying mechanisms are still currently unclear. Past studies have demonstrated that the PI3K/AKT/mTOR signaling pathway is an important pathway involved in myelination. In 2008, a study demonstrated hypermyelination in the CNS of Plp-Akt-DD mice, which are transgenic mice that express constitutively active Akt^25^. That study demonstrated that constitutively active Akt enhances CNS myelination. In 2009, they further showed activation of mTOR signaling and its downstream substrates (p70S6 kinase and S6 ribosomal protein) in these Plp-Akt-DD mice, and when mTOR signaling was inhibited with rapamycin, the hypermyelination was reduced^27^. Consistent with those results, Tyler *et al.* also showed that activation of mTOR signaling is important to oligodendrocyte differentiation *in vitro* and that phosphorylation of mTOR Ser 2448 correlates with myelination *in vivo*^29^. Furthermore, Goebbels *et al.* used conditional Pten mutant mice to demonstrate that the elevated levels of phosphatidylinositol 3,4,5-trisphosphate (PIP3) have promoting myelination in the CNS^26^. In 2014, Snaidero *et al.* further used an integrative approach that involved live imaging, electron microscopy and genetics to show that PI3K/AKT/mTOR signaling pathway is involved in the process of myelin membrane wrapping of CNS axons^28^. These studies confirm that the activated PI3K/AKT/mTOR signaling pathway contributes to the progression of myelination. In our research, we determined the proteins in PI3K/AKT/mTOR signaling pathway in the three experimental groups. The results showed no obvious differences in the total levels of AKT and m-TOR in the three groups, while the levels of p-Akt and p-mTOR are increased after LINGO-1 antibody treatment compared with EAE mice without treatment. The results imply that the LINGO-1 antibody may promote remyelination through the activated PI3K/AKT/mTOR signaling pathway.

In conclusion, we suggest that myelin damage in the PHC and fimbria-fornix may be the cause of the learning and memory impairment in EAE mice. The systemic administration of the LINGO-1 antibody improved the learning and memory ability of EAE mice. In addition, indicators of myelin damage improved, and the levels of proteins associated with neural structure and function was increased in the PHC after the six-week treatment. Furthermore, the LINGO-1 antibody may activate the PI3K/AKT/mTOR signaling pathway to promote remyelination. Taken together, our research demonstrated that LINGO-1 antagonism may be an effective approach to the treatment of the cognitive impairment of MS patients.

## Methods

### Animals

Adult (C57BL/6, ten-week-old, male) mice were purchased from the Animal Research Center of Shanghai Laboratory and were housed at 22 °C–24 °C. Food and water were available ad libitum. Animals were cared for in accordance with the National Institutes of Health Guidelines for Animal Care. All experimental procedures were approved by Jiang Su Animal Care and Use Committee in Southeast University.

### EAE

Ten-week-old C57BL/6 male mice were anesthetized by intraperitoneal injection of chloral hydrate (0.08 ml–0.1 ml of 10% chloral hydrate). EAE was induced by subcutaneous injection of an emulsion containing purified auto-antigen myelin oligodendrocyte glycoprotein (MOG) peptide, amino acids 35–55 (25 μg per mouse, GL Biochem, Shanghai, China), and mycobacterium tuberculosis (100 μg per mouse, BD) suspended in incomplete Freund’s adjuvant (sigma), as described. Then, the mice received 200 ng pertussis toxin (sigma) intraperitoneally (i.p.) on day 0 and day 2 post-immunization. The control mice were injected with the emulsion without MOG. The mice were monitored daily, and clinical disease severity was measured using the standard EAE grading scale. The standard grading scale ranges from 0 to 5:0, unaffected; 1, loss of tail tone; 2, limb weakness; 3, partial paralysis; 4, complete paralysis; and 5, moribund and/or death. Individual clinical scores were averaged across all animals per day, yielding a mean clinical disease index for each group.

### Behavioral Analyses

Before the behavioral tests, the animals were allowed to acclimate to their new environment for two days. The order of the behavior tests was as follows: the elevated plus maze, open field test, sucrose preference test (low-stimulation experiments), and the Morris water maze (high-stimulation one). The way to perform the elevated plus maze, open field test, sucrose preference test was provided in the supplementary information.

### Morris water maze

The water maze test was a best way to test spatial learning and memory ability and performed as previously described. Briefly, the maze consisted of a 1.2-m diameter circular pool filled with water (22 °C) that was made opaque by the addition of non-toxic, water-based white food coloring. A circular Plexiglas escape platform (10 cm in diameter) was located in the center of one of the quadrants of the pool. The experiment consisted of two phases including consecutive training days (four days or five days) and one detecting day. The animals underwent four trials over the training days with the platform submerged 1.5 cm below the surface of the water (60 s maximum trial duration; 20–30 min interval). The latency to reach the platform was analyzed to assess learning in the mice. On the last day, a probe test was performed in which the platform was removed and was out of the reach of the mice. The mice were tested with a single trial without the platform starting from the opposite quadrant of the platform for 60 s. The percentage of the distance in the target quadrant was measured to evaluate the memory ability.

### LINGO-1 antibody treatment

The LINGO-1 antibody was generated following the method previously described by Mi *et al.*^23^, but using the BALB/c strain of mice. ELISA assay demonstrated specific binding of LINGO-1 monoclonal antibody to LINGO-1 protein (Supplementary Fig. S7 online). For systemic drug delivery, we intraperitoneally injected 10 mg/kg antibody once every six days, and began on day 14, when the EAE mice began to display clinical symptoms. The mice of the control group and the non-pharmacologic treatment EAE group were administered 0.9% NaCl.

### Western blot

Mice were killed after the behavior tests. Mice from each group were deeply anesthetized with chloral hydrate and perfused transcardially with ice-cold 0.9% saline. The brains were dissected from the skulls, and six different brain areas, including the prefrontal cortex, cingulate, caudate putamen (striatum), fimbria-fornix, dorsal hippocampus and PHC, were dissected under an anatomical microscope based on their stereotaxic coordinates.

Tissues were processed for homogenization and sonication on ice in lysis buffer containing 50 mM Tris-base (pH7.4), 150 mM NaCl, 1% Triton X-100, 1% sodium deoxycholate, 0.1% SDS, sodium orthovanadate, sodium fluoride, EDTA, leupeptin, 0.5 mM sodium vanadate, 1% NP-40, 0.1 mM phenylmethylsulfonyl fluoride, 1  μg/mL aprotinin, and 1 μg/mL leupeptin supplemented with protease inhibitors. The extracts were then centrifuged at 13,000 × g for 20 minutes at 4  °C. The total amount of protein in the supernatant was determined with the bicinchoninic acid method using the Bio-Rad Protein Assay kit (Bio-Rad Laboratories, Hercules, CA, USA) according to the manufacturer’s instructions. Next, the supernatant was diluted 5:1 with sample buffer (6× concentrate; Beyotime, China), then the samples were heated for 5 minutes at 100 °C and stored at −20 °C. Equal amounts of protein (10 μg per lane) were resolved on 8%, 10% and 15% SDS-polyacrylamide gels. Following the electrophoresis, the proteins were transferred to Immobilon-P Transfer membranes (Millipore, Billerica, MA, USA). The membranes were probed overnight at 4 °C with the following antibodies and dilutions: anti-MBP (anti-rat monoclonal; 1:2000; Abcam), anti-dynein (anti-mouse monoclonal; 1:800; Abcam), anti-KLC (anti-rabbit polyclonal; 1:500; Santa), anti-NF200 (anti-rabbit polyclonal; 1:2000; Sigma), anti-phospho-Akt (Ser473) (anti-rabbit monoclonal; 1:2000; Cell Signaling Technology), anti-Akt (anti-rabbit monoclonal; 1:2000; Cell Signaling Technology), anti-phospho-mTOR (Ser 2448) (anti-rabbit polyclonal antibody; 1:500; Bioworld Technology), anti-mTOR (anti-rabbit polyclonal antibody; 1:500; Bioworld Technology), and anti-α-tubulin (anti-rabbit monoclonal; 1:2000; KeyGEN Biotech). After rinsing, the membranes were incubated with the secondary antibody conjugated with horseradish peroxidase (HRP) at dilutions of 1:5000 for 60 minutes at room temperature. Signals were detected using an enhanced chemiluminescence kit (ECL, Millipore, Billerica, MA, USA). The signal strength was quantified using a gel pro plus imaging analyzer. The average background density was subtracted, and the integral optical density values (IOD) were measured.

### Immunofluorescence

Mice were killed after the behavior tests. Mice from each group were deeply anesthetized with chloral hydrate and perfused transcardially with ice-cold 0.9% saline, followed by 4% paraformaldehyde. The brains were dissected from the skulls, post-fixed with 4% paraformaldehyde over night, followed by 10%, 20% and 30% sucrose solutions, each for at least 16 hours. Brain tissue was embedded in Tissue Freezing Medium (Leica, Germany), frozen at −80 °C and cut with a Leica microtome into 20-μm coronal sections. Frozen sections were used to analyze the expression of the myelin basic protein (MBP). Sections were incubated over night at 4 °C with primary antibodies: anti-MBP (anti-rat monoclonal; 1:200; Abcam). Following the incubation with primary antibodies, sections were washed and incubated for 2 h at room temperature with secondary antibody donkey Alexa Fluor 488 F(ab) anti-rat IgG. Images were captured from stained frozen sections using a fluorescence microscope equipped with 10×objectives. All images (RGB) were converted to grayscale. Mean Optical density (OD) value was quantified by computerized image analysis using Image J and Mean OD value = IOD/area.

### Statistical analysis

The clinical scores are displayed as the means ± SD and other data are presented as the means ± SEM. A one-tailed Student’s t-test or a one-way ANOVA with Tukey’s post hoc test was used to determine statistical significance. Dunn’s multiple-comparison was used for analysis of differences in the clinical progression of EAE among the three groups of mice. P < 0.05 was set as the cutoff for statistical significance. The statistical analyses were performed using GraphPad Prism 4 software and SPSS 18.

## Additional Information

**How to cite this article**: Sun, J.-J. *et al.* LINGO-1 antibody ameliorates myelin impairment and spatial memory deficits in experimental autoimmune encephalomyelitis mice. *Sci. Rep.* **5**, 14235; doi: 10.1038/srep14235 (2015).

## Supplementary Material

Supplementary Information

## References

[b1] Chiaravalloti N. D. & DeLuca J. Cognitive impairment in multiple sclerosis. The Lancet. Neurology 7, 1139–1151 (2008). 19007738 10.1016/S1474-4422(08)70259-X

[b2] Strober L. B. *et al.* Unemployment in multiple sclerosis: the contribution of personality and disease. Multiple sclerosis (Houndmills, Basingstoke, England) 18, 647–653 (2012). 10.1177/1352458511426735 22183935

[b3] D’Intino G. *et al.* Cognitive deficit associated with cholinergic and nerve growth factor down-regulation in experimental allergic encephalomyelitis in rats. Proceedings of the National Academy of Sciences of the United States of America 102, 3070–3075 (2005). 15710875 10.1073/pnas.0500073102 PMC548798

[b4] Rahn K. A. *et al.* Inhibition of glutamate carboxypeptidase II (GCPII) activity as a treatment for cognitive impairment in multiple sclerosis. Proceedings of the National Academy of Sciences of the United States of America 109, 20101–20106 (2012). 23169655 10.1073/pnas.1209934109 PMC3523869

[b5] Ziehn M. O., Avedisian A. A., Tiwari-Woodruff S. & Voskuhl R. R. Hippocampal CA1 atrophy and synaptic loss during experimental autoimmune encephalomyelitis, EAE. Laboratory investigation; a journal of technical methods and pathology 90, 774–786 (2010). 10.1038/labinvest.2010.6 PMC3033772 20157291

[b6] Funfschilling U. *et al.* Glycolytic oligodendrocytes maintain myelin and long-term axonal integrity. Nature 485, 517–521 (2012). 22622581 10.1038/nature11007 PMC3613737

[b7] Lee Y. *et al.* Oligodendroglia metabolically support axons and contribute to neurodegeneration. Nature 487, 443–448 (2012). 22801498 10.1038/nature11314 PMC3408792

[b8] Nave K. A. Myelination and support of axonal integrity by glia. Nature 468, 244–252 (2010). 21068833 10.1038/nature09614

[b9] McTigue D. M. & Tripathi R. B. The life, death, and replacement of oligodendrocytes in the adult CNS. Journal of neurochemistry 107, 1–19 (2008). 18643793 10.1111/j.1471-4159.2008.05570.x

[b10] Mifsud G., Zammit C., Muscat R., Di Giovanni G. & Valentino M. Oligodendrocyte pathophysiology and treatment strategies in cerebral ischemia. CNS neuroscience & therapeutics 20, 603–612 (2014). 24703424 10.1111/cns.12263 PMC6493108

[b11] Bartzokis G. Alzheimer’s disease as homeostatic responses to age-related myelin breakdown. Neurobiology of aging 32, 1341–1371 (2011). 19775776 10.1016/j.neurobiolaging.2009.08.007 PMC3128664

[b12] Butts B. D., Houde C. & Mehmet H. Maturation-dependent sensitivity of oligodendrocyte lineage cells to apoptosis: implications for normal development and disease. Cell death and differentiation 15, 1178–1186 (2008). 18483490 10.1038/cdd.2008.70

[b13] Fields R. D. White matter in learning, cognition and psychiatric disorders. Trends in neurosciences 31, 361–370 (2008). 18538868 10.1016/j.tins.2008.04.001 PMC2486416

[b14] Akbar N. *et al.* Diffusion tensor imaging abnormalities in cognitively impaired multiple sclerosis patients. The Canadian journal of neurological sciences. Le journal canadien des sciences neurologiques 37, 608–614 (2010). 21059506 10.1017/s0317167100010775

[b15] Fink F. *et al.* The association between California Verbal Learning Test performance and fibre impairment in multiple sclerosis: evidence from diffusion tensor imaging. Multiple sclerosis (Houndmills, Basingstoke, England) 16, 332–341 (2010). 10.1177/1352458509356367 20150400

[b16] Mesaros S. *et al.* Diffusion tensor MRI tractography and cognitive impairment in multiple sclerosis. Neurology 78, 969–975 (2012). 22377806 10.1212/WNL.0b013e31824d5859

[b17] Dutta R. *et al.* Demyelination causes synaptic alterations in hippocampi from multiple sclerosis patients. Annals of neurology 69, 445–454 (2011). 21446020 10.1002/ana.22337 PMC3073544

[b18] Geurts J. J. *et al.* Extensive hippocampal demyelination in multiple sclerosis. Journal of neuropathology and experimental neurology 66, 819–827 (2007). 17805012 10.1097/nen.0b013e3181461f54

[b19] Patti F. Treatment of cognitive impairment in patients with multiple sclerosis. Expert opinion on investigational drugs 21, 1679–1699 (2012). 22876911 10.1517/13543784.2012.716036

[b20] Mi S., Sandrock A. & Miller R. H. L. INGO-1 and its role in CNS repair. The international journal of biochemistry & cell biology 40, 1971–1978 (2008). 18468478 10.1016/j.biocel.2008.03.018

[b21] Mi S. *et al.* LINGO-1 negatively regulates myelination by oligodendrocytes. Nature neuroscience 8, 745–751 (2005). 15895088 10.1038/nn1460

[b22] Lee X. *et al.* NGF regulates the expression of axonal LINGO-1 to inhibit oligodendrocyte differentiation and myelination. The Journal of neuroscience : the official journal of the Society for Neuroscience 27, 220–225 (2007). 17202489 10.1523/JNEUROSCI.4175-06.2007 PMC6672289

[b23] Mi S. *et al.* LINGO-1 antagonist promotes spinal cord remyelination and axonal integrity in MOG-induced experimental autoimmune encephalomyelitis. Nature medicine 13, 1228–1233 (2007). 10.1038/nm1664 17906634

[b24] Maday S., Twelvetrees A. E., Moughamian A. J. & Holzbaur E. L. Axonal transport: cargo-specific mechanisms of motility and regulation. Neuron 84, 292–309 (2014). 25374356 10.1016/j.neuron.2014.10.019 PMC4269290

[b25] Flores A. I. *et al.* Constitutively active Akt induces enhanced myelination in the CNS. The Journal of neuroscience : the official journal of the Society for Neuroscience 28, 7174–7183 (2008). 18614687 10.1523/JNEUROSCI.0150-08.2008 PMC4395496

[b26] Goebbels S. *et al.* Elevated phosphatidylinositol 3,4,5-trisphosphate in glia triggers cell-autonomous membrane wrapping and myelination. The Journal of neuroscience : the official journal of the Society for Neuroscience 30, 8953–8964 (2010). 20592216 10.1523/JNEUROSCI.0219-10.2010 PMC6632897

[b27] Narayanan S. P., Flores A. I., Wang F. & Macklin W. B. Akt signals through the mammalian target of rapamycin pathway to regulate CNS myelination. The Journal of neuroscience: the official journal of the Society for Neuroscience 29, 6860–6870 (2009). 19474313 10.1523/JNEUROSCI.0232-09.2009 PMC2757755

[b28] Snaidero N. *et al.* Myelin membrane wrapping of CNS axons by PI(3,4,5)P3-dependent polarized growth at the inner tongue. Cell 156, 277–290 (2014). 24439382 10.1016/j.cell.2013.11.044 PMC4862569

[b29] Tyler W. A. *et al.* Activation of the mammalian target of rapamycin (mTOR) is essential for oligodendrocyte differentiation. The Journal of neuroscience : the official journal of the Society for Neuroscience 29, 6367–6378 (2009). 19439614 10.1523/JNEUROSCI.0234-09.2009 PMC2827328

[b30] Politis M. *et al.* Increased PK11195 PET binding in the cortex of patients with MS correlates with disability. Neurology 79, 523–530 (2012). 22764258 10.1212/WNL.0b013e3182635645 PMC3413767

[b31] Achiron A., Chapman J., Tal S., Bercovich E. & Gil H. Superior temporal gyrus thickness correlates with cognitive performance in multiple sclerosis. Brain structure & function 218, 943–950 (2013). 22790785 10.1007/s00429-012-0440-3

[b32] Huitinga I. *et al.* Hypothalamic lesions in multiple sclerosis. Journal of neuropathology and experimental neurology 60, 1208–1218 (2001). 11764093 10.1093/jnen/60.12.1208

[b33] Kern K. C. *et al.* Fornix damage limits verbal memory functional compensation in multiple sclerosis. NeuroImage 59, 2932–2940 (2012). 22001266 10.1016/j.neuroimage.2011.09.071

[b34] Levy Barazany H. *et al.* Brain MRI of nasal MOG therapeutic effect in relapsing-progressive EAE. Experimental neurology 255, 63–70 (2014). 24552689 10.1016/j.expneurol.2014.02.010

[b35] Vorhees C. V. & Williams M. T. Morris water maze: procedures for assessing spatial and related forms of learning and memory. Nature protocols 1, 848–858 (2006). 17406317 10.1038/nprot.2006.116 PMC2895266

[b36] Fields R. D. Neuroscience. Myelin–more than insulation. Science (New York, N.Y.) 344, 264–266 (2014). 10.1126/science.1253851 PMC5017201 24744365

[b37] Haroutunian V. *et al.* Myelination, oligodendrocytes, and serious mental illness. Glia 62, 1856–1877 (2014). 25056210 10.1002/glia.22716

[b38] Nave K. A. Myelination and the trophic support of long axons. Nature reviews. Neuroscience 11, 275–283 (2010). 10.1038/nrn2797 20216548

[b39] Pepinsky R. B. *et al.* Exposure levels of anti-LINGO-1 Li81 antibody in the central nervous system and dose-efficacy relationships in rat spinal cord remyelination models after systemic administration. The Journal of pharmacology and experimental therapeutics 339, 519–529 (2011). 21807883 10.1124/jpet.111.183483

