# Supplementary material for: LINGO-1 antibody ameliorates myelin impairment and spatial memory deficits in experimental autoimmune encephalomyelitis mice
Source: Sci Rep. 2015 Sep 18;5:14235. doi: 10.1038/srep14235 (PMC4585639; doi:10.1038/srep14235)
Supplement: Supplementary Information [file srep14235-s1.pdf]

LINGO-1 antibody ameliorates myelin impairment and spatial memory deficits in experimental autoimmune encephalomyelitis mice

Jun-Jun Sun[1,+] , Qing-Guo Ren[1,*,+] , Lin Xu[2, 3], Zhi-Jun Zhang[1,*]

1Department of Neuropsychiatry, Affiliated ZhongDa Hospital, School of Medicine, Southeast University, Nanjing 210009, Jiangsu, China.

2Key Laboratory of Animal Models and Human Disease Mechanisms, Chinese Academy of Sciences & Yunnan Province, Kunming Institute of Zoology, Kunming650223, Yunnan, China

3Graduate School of Chinese Academy of Sciences, Beijing100049, China

*corresponding.author@email.example

+these authors contributed equally to this work

Correspondence should be addressed to:

Qing-Guo Ren Ph.D.; Prof. Zhi-Jun Zhang

Tel: +86-25-83262255

Fax: +86-25-83262255

Email: renqingguo1976@163.com

zhijunzhang@seu.edu.cn

Supplementary methods

The elevated plus maze (EPM)

The elevated plus maze (EPM) is a widely used test for anxiety behavior in rodents. The EPM test was conducted as previously described. Briefly,

the mice were placed in the center of the maze facing an open arm and were allowed to freely explore the EPM for five minutes. A mouse that placed all four paws onto the arm was considered to be in it, and otherwise was considered to be in the center of the maze. The percentage (%) of open arm distances and the percentage (%) of time spent on the open arms were rcorded to measure the anxiety of mice. Decreased open arm activities indicate increased anxiety levels in the EPM. Between each trial, the maze was wiped clean with a damp sponge and dried with paper towels.

Open field test

The open field test is an experiment that is used to assess general locomotor activity levels and anxiety in rodents. Each mouse was placed in the center of the open field apparatus (50 cm x 50 cm x 60 cm) and can move freely for 5 min. The average speed and time/distance in the center was recorded to measure the locomotor activity and anxiety levels. Between each trial, the maze was wiped clean with a damp sponge and dried with paper towels.

Sucrose preference test

The sucrose preference test is used to test the level of anhedonia in mice. The mice were habituated to 2% sucrose solution for one day prior to the start of the experiment. On the test day, the mice were housed singly with ad libitum food and two bottles—one with water and the other with 2%

sucrose solution—for 24 hours. The bottles were reversed halfway through the time to avoid a side preference. The weights of the two bottles were recorded to calculate the sucrose consumption. The preference for the sucrose solution was calculated as a percentage of total liquid consumed. The sucrose preference rate was calculated using the following formula: sucrose preference rate = sucrose consumption / (water consumption + sucrose consumption) × 100%.

Supplementary Figure legends

Supplementary Fig. S1. No difference in the open field test between the EAE and control groups both in the early and late stages of disease (a1) In the early stage, the speed of the EAE mice in the open field test was similar to the controls. (a2 and a3) And the percentage of distance/time in the central area of EAE mice was also close to that of controls. (b1, b2 and b3) Similarly, in the late stage, no significant difference was observed in the speed and the percentage of distance/time in the central area between the two groups.

Supplementary Fig. S2. No difference in the elevated plus maze between the EAE and control groups both in the early and late stages of disease (a1 and b1) The percentage of distance on the open arms in the EAE mice was closed to that of controls both in the early and late stages of disease. (a2 and b2) And the percentage of time spent on the open arms displayed

no difference in the EAE mice and controls (P>0.05) both in the two stages.

Supplementary Fig. S3. No difference in the sucrose preference test between the EAE mice and controls both in the early and late stages of disease (a and b) The sucrose preference ratio of the EAE mice was similar to that of controls both in the early and late stages of disease.

Supplementary Fig. S7. LINGO-1 specific antibody binds to LINGO-1 protein (a) LINGO-1 antibody is one of monoclonal antibodies. ELISA assay was used to demonstrate specific binding of LINGO-1 antibody to LINGO-1 protein and it also showed that LINGO-1 antibody was not binging to dC-his-tagged protein, which is the negative control protein. (b) LINGO-1 expression was detected in mice tissue lysates with LINGO-1 antibody through western blot.

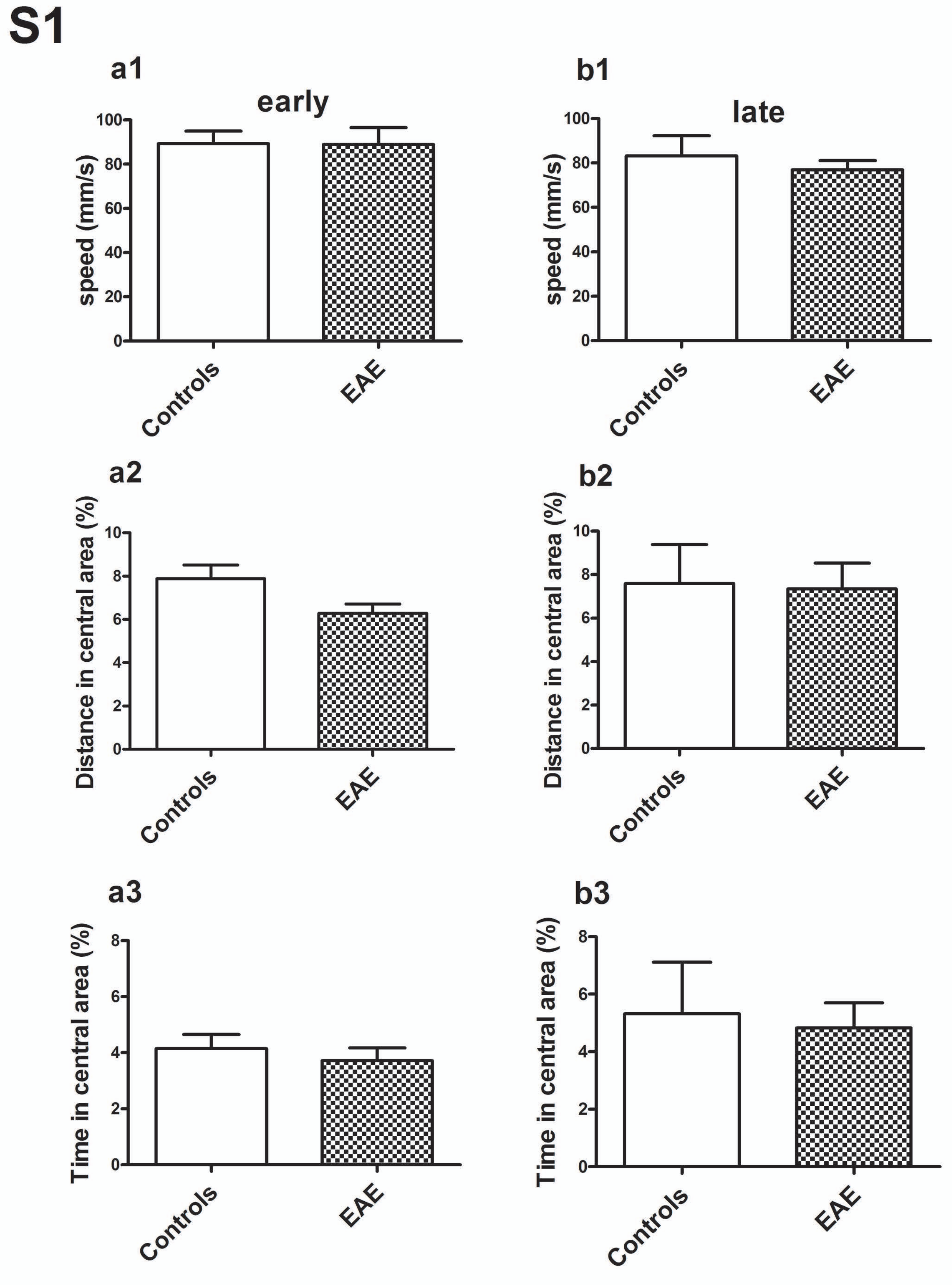
S1
a1
early
speed (mm/s)
0
20
40
60
80
100
Controls
EAE
b1
late
speed (mm/s)
0
20
40
60
80
100
Controls
EAE
a2
Distance in central area (%)
0
2
4
6
8
10
Controls
EAE
b2
Distance in central area (%)
0
2
4
6
8
10
Controls
EAE
a3
Time in central area (%)
0
2
4
6
8
Controls
EAE
b3
Time in central area (%)
0
2
4
6
8
Controls
EAE

S2

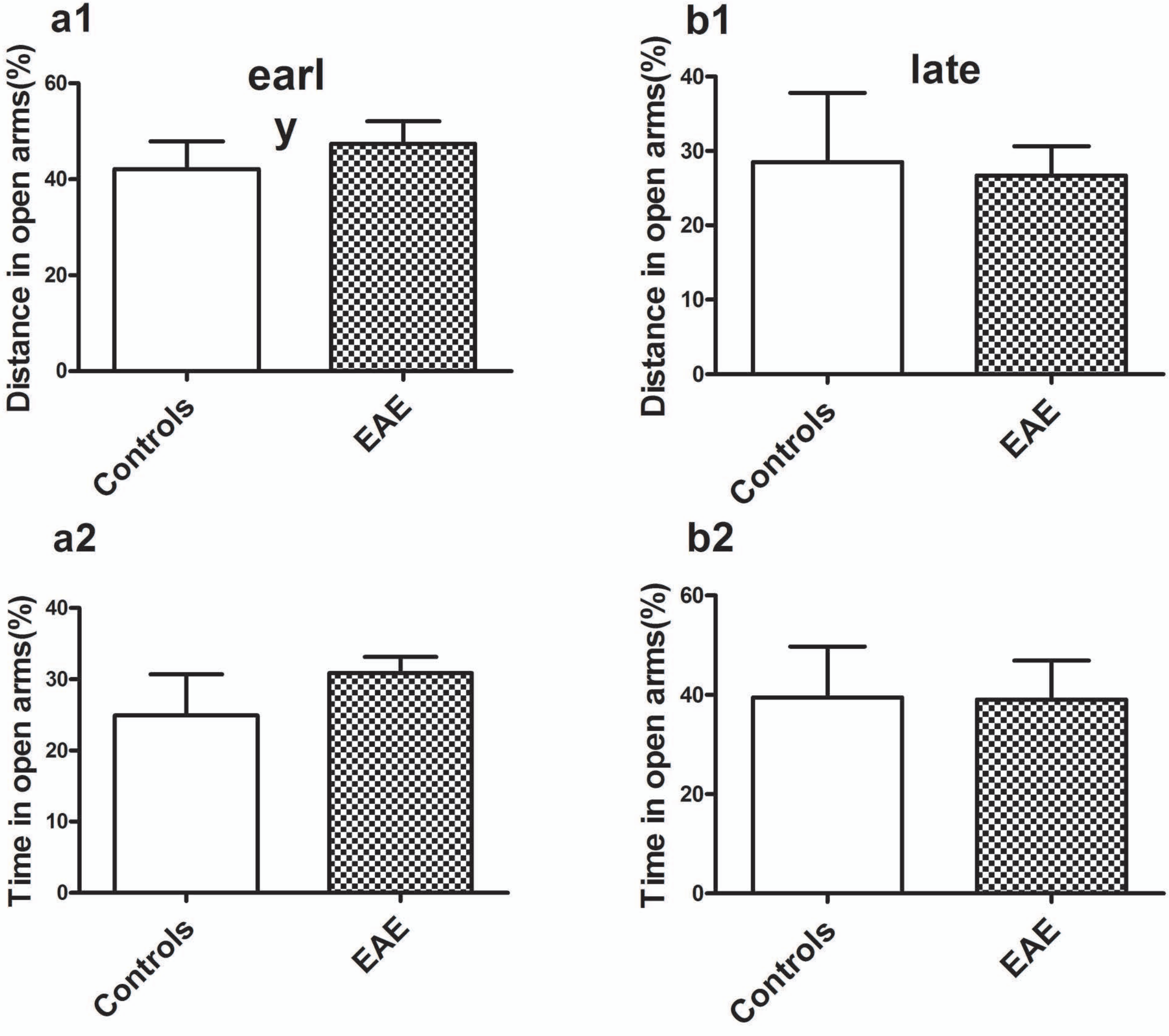

a1
earl
y
Distance in open arms(%)
60
40
20
0
Controls
EAE
b1
late
Distance in open arms(%)
40
30
20
10
0
Controls
EAE
a2
Time in open arms(%)
40
30
20
10
0
Controls
EAE
b2
Time in open arms(%)
60
40
20
0
Controls
EAE

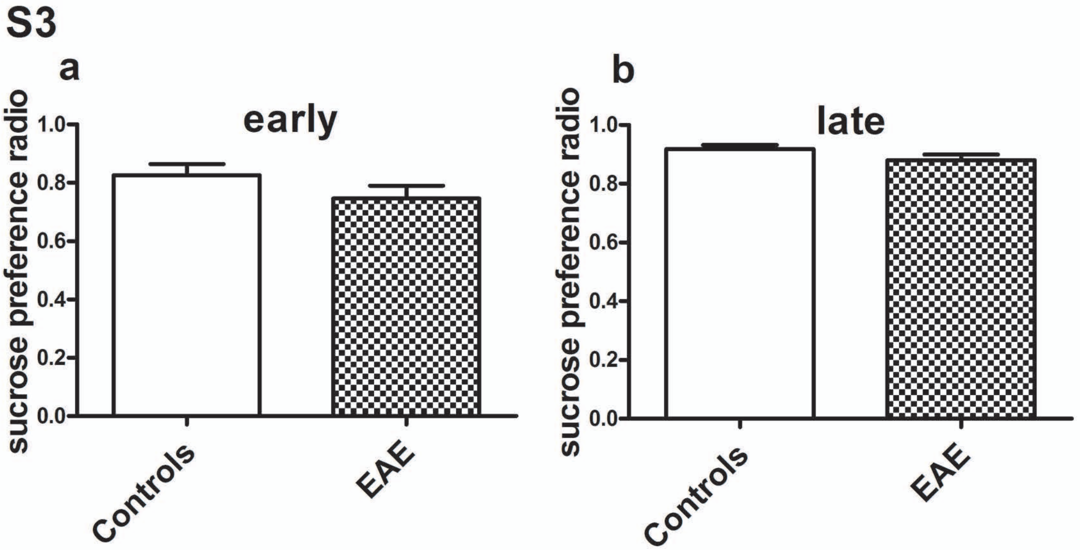
S3
a
early
sucrose preference radio
1.0
0.8
0.6
0.4
0.2
0.0
Controls
EAE
b
late
sucrose preference radio
1.0
0.8
0.6
0.4
0.2
0.0
Controls
EAE

S4 Full-length blots of Figure 2 in the main text

Fig 2(a)

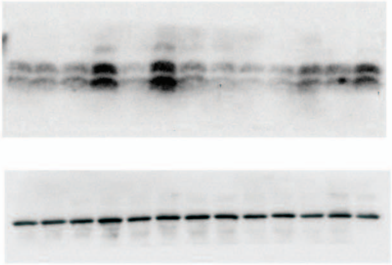

Fig 2(b)

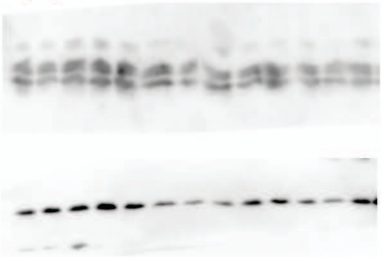

Fig 2(c)

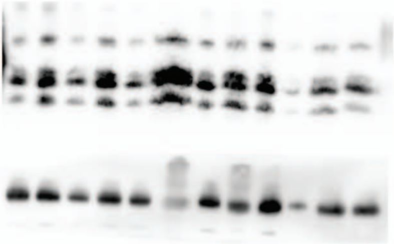

Fig 2(d)

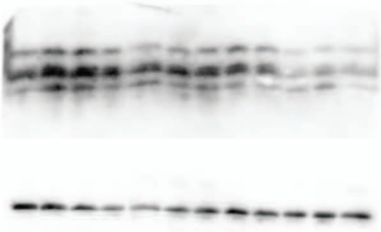

Fig 2(e)

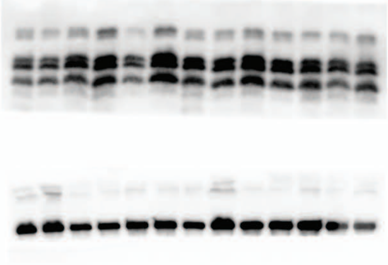

Fig 2(f)

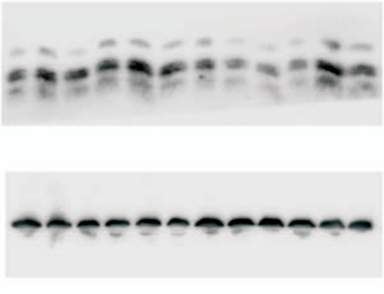

S5 Full-length blots of Figure 4 in the main text

Fig 4(a)

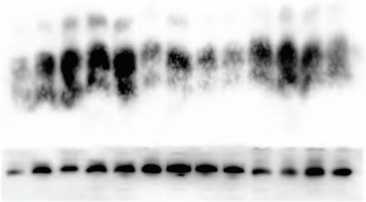

Fig 4(b)

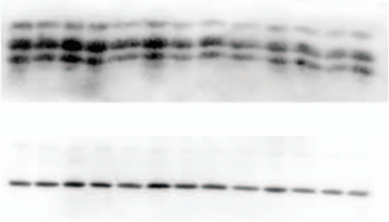

Fig 4(c)

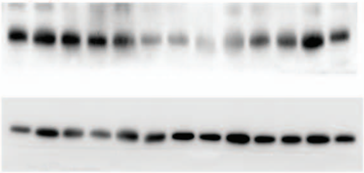

Fig 4(d)

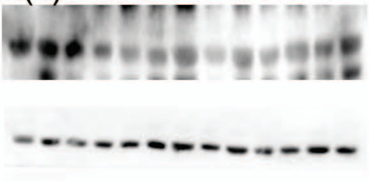

Fig 4(e)

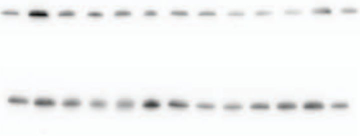

Fig 4(f)

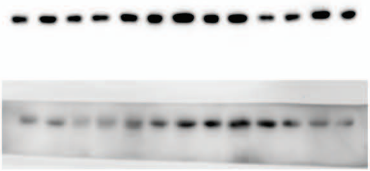

Fig 4(g)

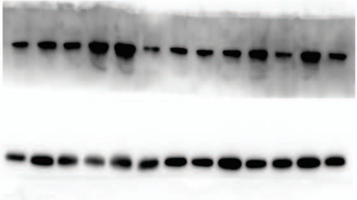

Fig 4(h)

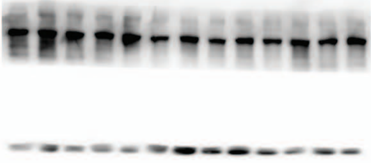

S6 Full-length blots of Figure 5 in the main text

Fig 5(a)

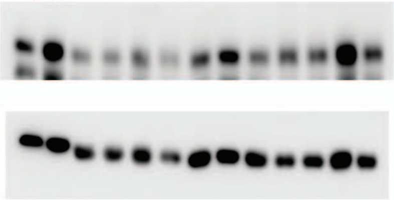

Fig 5(b)

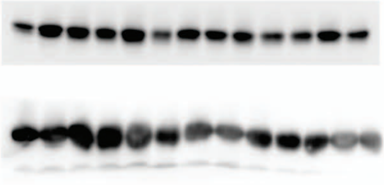

Fig 5(c)

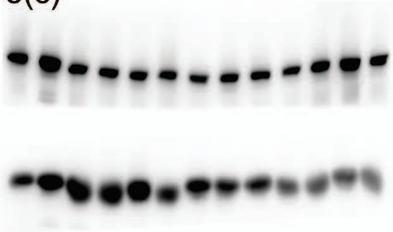

Fig 5(d)

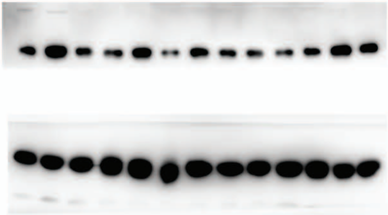

S7

(a)

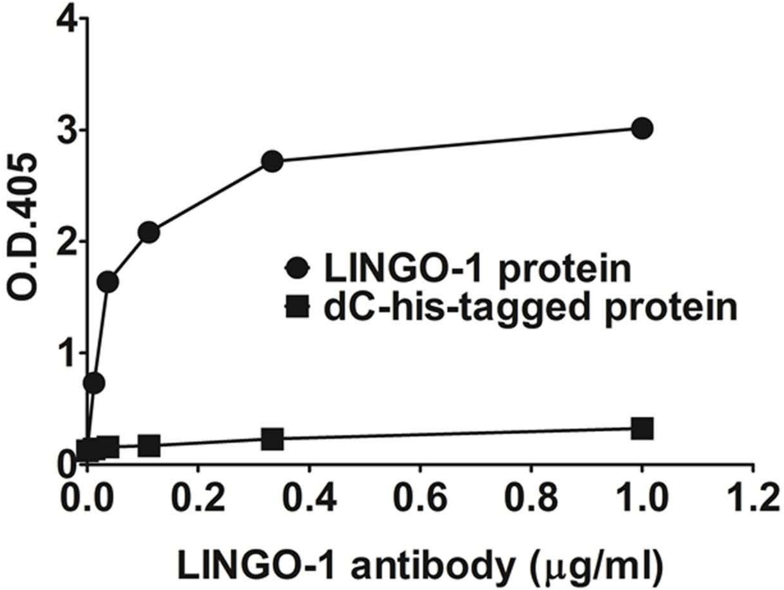
O.D.405
4
3
2
1
0
LINGO-1 protein
dC-his-tagged protein
0.0
0.2
0.4
0.6
0.8
1.0
1.2
LINGO-1 antibody (μg/ml)

(b)

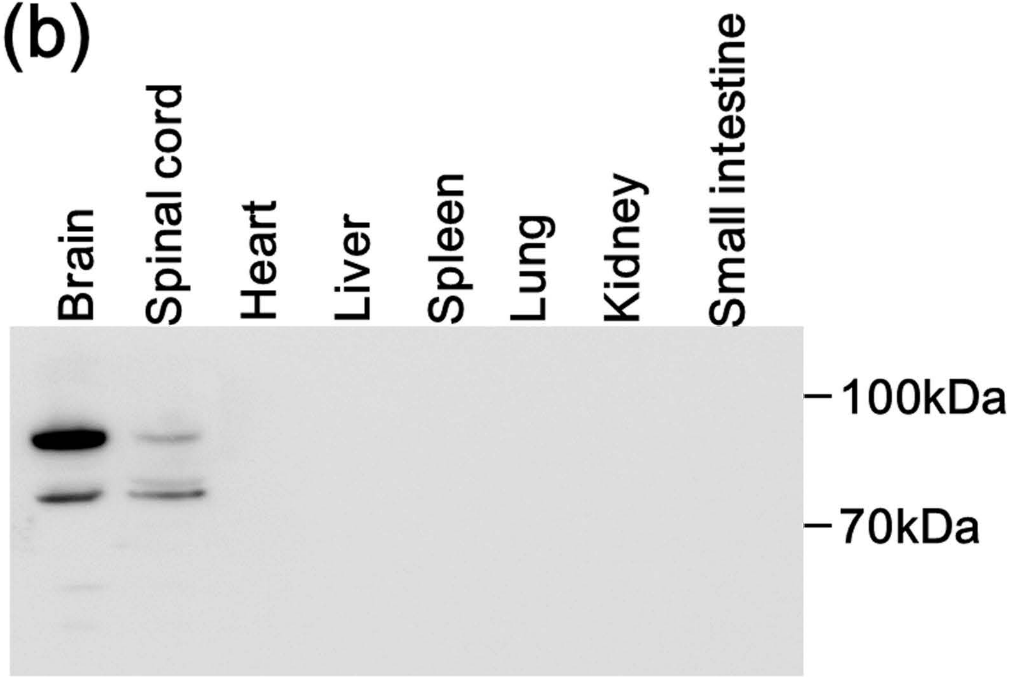
Brain
Spinal cord
Heart
Liver
Spleen
Lung
Kidney
Small intestine
100kDa
70kDa
